# Interfacial Polarization-Driven Dielectric–Magnetic Synergy in Vitrimeric f-MWNT/ZnO Composites: Effect of MWNT Functionalization

**DOI:** 10.3390/polym18111374

**Published:** 2026-06-01

**Authors:** Nehal Kaushik, Madhuri Surya, Divyanshi Nautiyal, Rajkumar Patel, Sravendra Rana

**Affiliations:** 1 School of Engineering, University of Petroleum & Energy Studies (UPES), Dehradun 248007, Uttarakhand, India; 2 Integrated Science and Engineering Division, Underwood International College, Yonsei University, Incheon 21983, Republic of Korea

**Keywords:** electromagnetic wave absorption, vitrimers, acid functionalization, self-healing, X-band absorption

## Abstract

The rise in electromagnetic radiation has created a dire need for the development of sheer absorbing composites with tunable dielectric and magnetic responses. With interfacial engineering in carbonaceous systems, high absorption efficiency and sheerness can be obtained in the X-band region. In this work, acid-functionalized carbon nanotube/ZnO (f-MWNT/ZnO) composites have been developed and investigated to understand the effect of functionalization on electromagnetic response. Permittivity data revealed a stronger frequency-dependent response in f-MWNT/ZnO, ascribed to polarization losses induced by oxygen-containing functional groups. Furthermore, dielectric loss and Cole–Cole plots indicated numerous Debye relaxation processes in combination with Maxwell–Wagner–Sillars polarizations. Permeability measurements signify distinct peaks for f-MWNT/ZnO attributed to exchange and natural resonance; however, the pristine carbon nanotube-derived (MWNT/ZnO) composite exhibits a weaker response. Stemming from the synergy of dielectric–magnetic interactions and improved impedance matching, the f-MWNT/ZnO composite with a thickness of 1.9 mm achieved an RL of −12.7 dB, corresponding to a ~94% absorption efficiency at 12.3 GHz. Additionally, the composite exhibited autonomous self-healing, enabling the reintegration of two separated segments at 70 °C for 40 min. The findings highlight the critical role of functionalization in tailoring interfacial characteristics and enhancing absorption performance.

## 1. Introduction

As electromagnetic (EM) waves have become more prevalent in everyday life, healthcare, radar systems and military applications, there has been an increase in the demand for EM wave absorption materials throughout recent years. In particular, microwave absorbers (MAs) are important as they serve in military applications to disguise the movement of various strategic targets such as airplanes, tanks or ships by decreasing their radar cross section (RCS) [1,2]. For practical applications, MAs must be mechanically robust, lightweight, cost-effective, thin and have a broad absorption frequency band. Achieving all of these qualities in a single absorber is a difficult task; thus, researchers are looking at different manufacturing processes to achieve the desired outcomes [3]. Anciently, the most significant problem faced was impedance mismatch caused by a strong dielectric or magnetic response, which increased reflection on the air–absorber interface and reduced absorption [4]. As a result, dielectric–magnetic synergy was used so the benefits of both features could be gained. However, due to their high density, corrosion susceptibility and secondary EM radiation, the use of magnetic metals is still avoided [5]. To achieve the aforementioned prerequisites, the use of multiwalled carbon nanotubes (MWNTs) has increased dramatically due to their high aspect ratio, superior conductivity and dissipation qualities [6,7,8]. Furthermore, the defects and unsaturated bonds on their surface can aid interfacial polarization and multiple scattering [9]. MWNTs are typically coupled with other metallic nanoparticles to prevent reflection dominance and improve performance. Metallic oxides such as ZnO, Al_2_O_3_, TiO_2_ and others have recently received attention because of their high thermal conductivities, high dielectric constants and outstanding strength and modulus [10,11,12]. These metallic oxides are utilized as a secondary reinforcer in polymeric matrices to improve complex permittivity and, ultimately, the overall absorption efficiency of MAs.

It is generally said that rational microstructure designs can improve absorber performance and efficiency by enhancing polarization, conduction and resonance losses [13,14,15]. Heterointerface engineering has been shown to be a viable way to improve loss mechanisms since it generates multiple interfaces in absorbers. For instance, Fe_3_O_4_/MWNT [16], TiO_2_@Fe_3_O_4_@PPy [17] and FeSiAl@Al_2_O_3_@SiO_2_ composites have good absorption performance owing to the many interfaces formed by the components. Another technique for increasing the number of hetero-surfaces is to dope or functionalize the components to improve their characteristics. In recent years, efforts have focused on functionalizing groups onto/into the dielectric component to generate synergy between the dielectric and magnetic losses [18,19,20,21,22]. For instance, Wen and coworkers [20] synthesized several metal-doped MWNTs and studied their absorption behavior, revealing improved performance due to the synergy of complex permeability and permittivity values. Similarly, Che and coworkers [23] encapsulated Fe particles in MWNTs and observed a better response in magnetic effects due to Fe confinement.

During on-ground application, the material will wear and tear, causing the mechanical strength to degrade. There is also a risk of wave leakage, which can render the concealment purpose ineffective [24]. The usage of vitrimeric polymer matrices is an excellent solution to both of these problems. Vitrimers have the innate capacity to self-heal in response to stimuli such as pH, temperature or irradiation, which can extend coating lifespan and the durability of coatings [25]. Self-healing can be achieved via a variety of reversible bonding techniques like Schiff base [26], transesterification [27], disulfide bond exchange [28,29,30], click chemistry [31,32,33] and many more [34,35,36,37]. Furthermore, vitrimeric matrices have excellent mechanical strength, endurance and malleability which is quite advantageous in applications like stealth.

Herein, to address the need for sheer and healable MAs, we report the development of an acid-functionalized carbon nanotube/ZnO composite (f-MWNT/ZnO), engineered for X-band absorption. Contrasting an f-MWNT/ZnO composite with pristine counterparts (MWNT/ZnO), we demonstrate how oxygen-containing functional moieties can serve as crucial sites for enhancing polarization losses and impedance matching. The work reveals that f-MWNT/ZnO composites exhibit synergy, Debye relaxation, Maxwell–Wagner–Sillars polarization and magnetic losses to achieve an absorption efficiency of ~94% at a thickness of 1.9 mm. Importantly, we propose self-healable composites that reintegrate at 70 °C in 40 min, offering a robust solution to the mechanical degradation faced by composites in demanding environments. This work strategically provides a framework for harnessing interfacial characteristics to achieve a strong absorption response in addition to material longevity.

## 2. Experimental Details

### 2.1. Materials

Carbon nanotubes (MWNTs) with an outer diameter of 30–50 nm and a length of 10–30 µm and acid-functionalized carbon nanotubes (MWNT-COOH) with an OD of 10–20 nm and a length of 10–30 µm were sourced from SRL chemicals. ZnO nanoparticles with a size of ≤50 nm were procured from Sigma Aldrich (Bangalore, India). Thiol crosslinker Pentaerythritol tetrakis (3-mercaptopropionate) and a tin-based catalyst were also sourced from Sigma Aldrich. The solvents required for preparation and processing were of analytical grade and procured from well-established commercial sources.

### 2.2. Synthesis of Vitrimeric Composite

**Figure 1:**
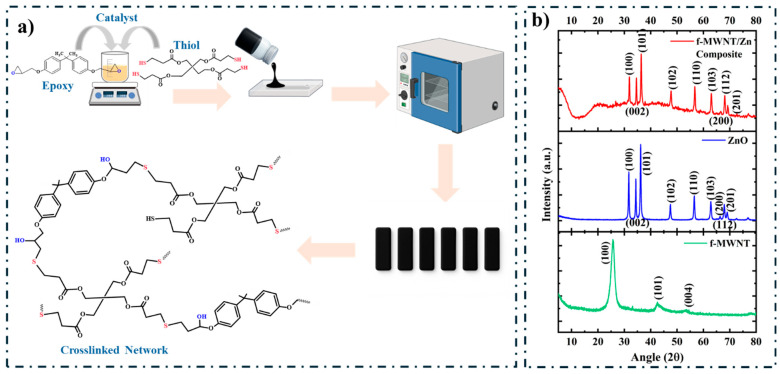
(**a**) Fabrication schematics of f-MWNT/ZnO and MWNT/ZnO vitrimeric composites; (**b**) XRD analysis of components and f-MWNT/ZnO vitrimeric composite.

Uniform distribution of filler components is a prerequisite to harness the properties of the composites to the fullest. Hence, 4 wt% of carbon nanotubes were first sonicated in ethanol before being magnetically stirred for 1 h to ensure uniform dispersion. After that, 20 wt% of ZnO was added to the nanotube suspension, succeeded by stirring for an additional 1 h at 60 °C. The temperature facilitates proper mixing of ZnO and nanotubes and gradual evaporation of ethanol. After complete dispersion, BADGE (epoxy) was added into the f-MWNT/ZnO slurry and stirred continuously till complete ethanol evaporation. The thiol-curing agent was then added along with the catalyst and the mixture was stirred continuously till a homogeneous phase had developed. As the reaction progressed, the viscosity increased, and after achieving pourable consistency, the mixture was casted into the PTFE moulds and kept in a vacuum oven for curing at 80 °C for 4 h and 120 °C for 2 h (as shown in Figure 1a).

### 2.3. Characterization

Preliminary structural confirmation of the composite and individual components was performed using X-ray Diffraction (XRD) with the Bruker D8 model Advance Eco Diffractometer and CuKα radiation, Bruker Corporation, Karlsruhe, Germany. Surface morphological images were captured using Tescan MIRA SEM, TESCAN group, Brno, Czech Republic. The electromagnetic response of the composites with l*b of 22.86*10.16 mm at various thicknesses was recorded using a Vector Network Analyzer (VNA) N5320C from Agilent Technologies, Santa Clara, CA, USA. The waveguide kit 11164A was used to measure the complex parameters in the X-band. Python code (Python 3.12.4) was used to compute different parameters from permeability and permittivity data in Jupyter Notebook 7.0.8. Thermo-mechanical analysis was performed by using a TA-Q400em thermo-mechanical analyzer (TA Instruments, Hüllhorst, Germany) in three-point bending mode with a nitrogen gas flow rate of 50 mL/min and an applied force of 0.02 N.

## 3. Results and Discussion

The XRD peaks of f-MWNT, ZnO and f-MWNT/ZnO composites are shown in Figure 1b; f-MWNTs exhibit two primary peaks at 25° and 42°, which align well with JCPDS No. 96-101-1061 and represent the interlayer spacing of the graphitic centres and the in-plane periodicity of the carbon [38]. The sharp diffraction peaks at 31°, 34°, 36°, 47°, 56°, 62°, 66°, 67° and 69° correspond to the (100), (002), (101), (102), (110), (103), (200), (112) and (201) planes, confirming the hexagonal wurtzite structure of the ZnO nanoparticles and agreeing with JCPDS data 36-1451 [39]. Expectedly, the composite (f-MWNT/ZnO) diffraction pattern has the dominant peaks of ZnO nanoparticles due to its superior scattering phase, as ZnO is present in a larger concentration (20 wt%). However, a small reflection peak of f-MWNTs is visible in the composite diffraction pattern, confirming the presence of f-MWNTs as well. The concurrent appearance of peaks of both f-MWNTs and ZnO signifies the successful formation of the composite without altering the basic structures of the individual components.

**Figure 2:**
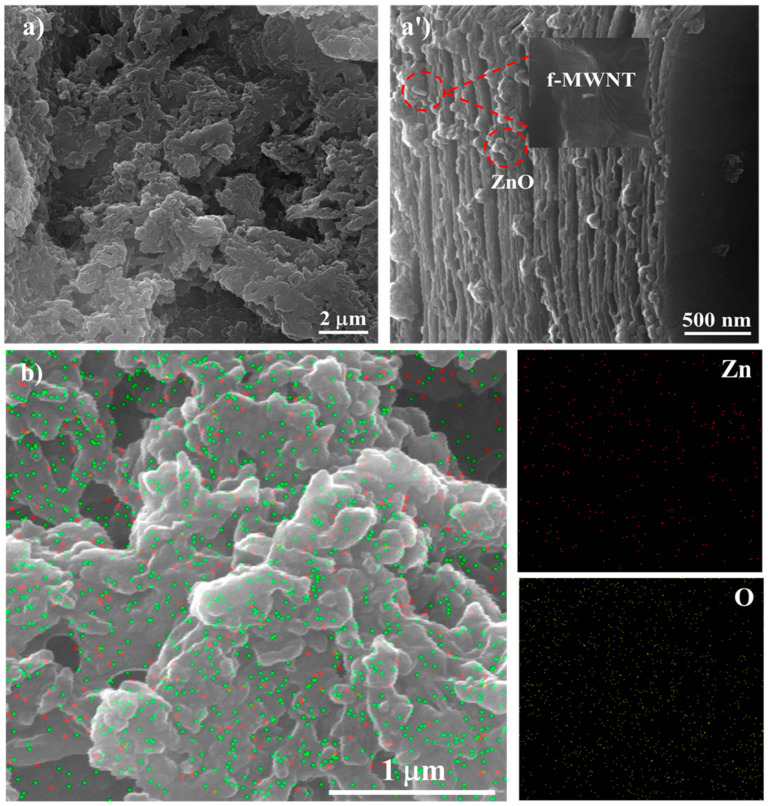
(**a**,**a′**) SEM images of f-MWNT/ZnO and (**b**) EDX mapping of f-MWNT/ZnO vitrimeric composites.

Surface morphological images of the f-MWNT/ZnO composite in Figure 2a reveal a dense interconnected network where ZnO and nanotubes are effectively dispersed. Higher-magnification images in Figure 2a′ demonstrate that ZnO particles are evenly adorned onto the layered structure, while f-MWNTs appear well dispersed, probably due to oxygen functional groups facilitating interfacial bonding [2,12]. Figure 2b shows EDX mapping of the f-MWNT/ZnO composite and validates the uniform distribution of the particles throughout the area. Such synchronized distribution of dielectric and conductive fillers generates multiple relaxation centers, leading to enhanced absorption performance. Additionally, Figure S1 shows the SEM image and EDX mapping of the MWNT/ZnO composite, revealing a well-dispersed composite network with no signs of agglomeration. As a result, it is clear that both composites are well dispersed and have no cluster formations; hence, the difference in the properties solely arises from the action of functionalization.

**Figure 3:**
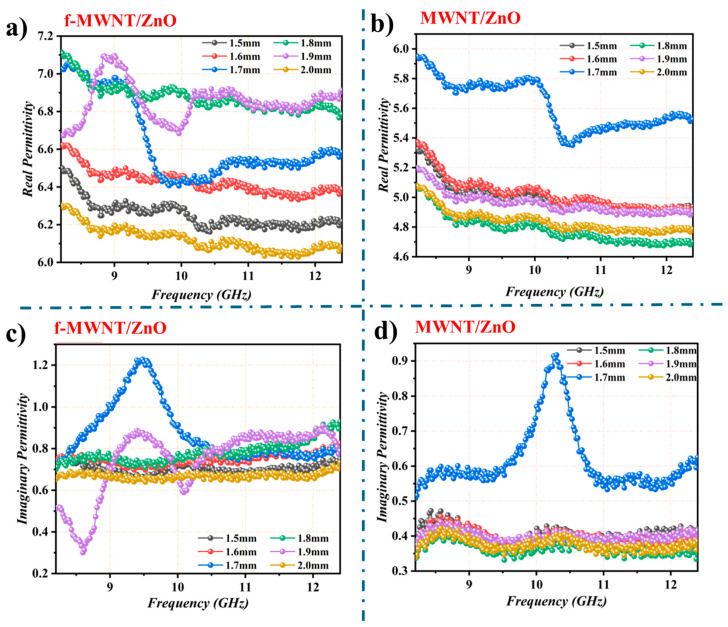
Real permittivity curves of (**a**) f-MWNT/ZnO and (**b**) MWNT/ZnO vitrimeric composites. Imaginary permittivity curves of (**c**) f-MWNT/ZnO and (**d**) MWNT/ZnO vitrimeric composites.

**Figure 4:**
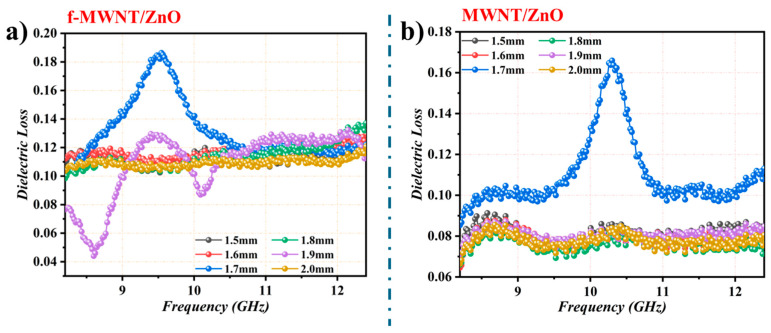
Dielectric loss curves of (**a**) f-MWNT/ZnO and (**b**) MWNT/ZnO vitrimeric composites.

The overall absorption performance of a composite is a direct reflection of the real (ε′, µ′) and imaginary components (ε″, µ″) of the permeability and permittivity values. Hence, the frequency-reliant behavior of the f-MWNT/ZnO and MWNT/ZnO composites is computed in the X-band with thicknesses from 1.5 mm to 2.0 mm. Figure 3a,b depict ε′, the real part of the permittivity, which denotes the material’s ability to store the electrical component of the electromagnetic wave [40]. Notably, the f-MWNT/ZnO composites exhibit higher ε′ values (between 6–7.1) compared to the MWNT/ZnO composites (between 4.6–6), which is due to the enhanced polarization ability of the acid-functionalized nanotubes. However, both compositions display a downward trend in the values as the frequency is increased, suggesting a lag of polarization in a rapidly alternating electric field [41]. This further validates the enhanced polarization of f-MWNT/ZnO composites as the dip is greater, especially for the 1.7 mm and 1.9 mm composites. Conversely, Figure 3c,d depict ε’’, the dissipation ability of the composite. Prominent peaks are observed for 1.7 mm and 1.9 mm samples at 9.5 GHz for f-MWNT/ZnO and at 10.3 GHz for 1.7 mm MWNT/ZnO composites, exactly at the frequency point where there is a dip in ε′. To further quantify the electric component dissipation of the composites, the dielectric loss tangent (tan δ_e_ = ε″/ε′) was evaluated (Figure 4). A similar trend in frequency-reliant behavior was observed, however, with superiority in the acid-functionalized system. For 1.7 mm and 1.9 mm f-MWNT/ZnO composite, higher loss tangents with fluctuations were observed, as shown in Figure 4a. The observed resonance peaks are probably due to the oxygen-rich functional groups (-OH, -COOH) introduced on the surface of nanotubes. These groups aid polarization by creating numerous interfaces and enhancing the contact between ZnO particles and nanotubes [42,43]. The obtained dissipation behavior aligns with the Maxwell–Wagner–Sillars effect as f-MWNT/ZnO composites with larger heterogeneous surfaces show greater dissipation in contrast to MWNT/ZnO composites, which show a flatter response due to a lack of active polarization sites (Figure 4b) [44]. This polarization response arises due to differences in the conductivity and permittivity of the composites’ components, generating interfacial polarization effects around the heterogeneous interfaces formed [45].

Furthermore, Cole–Cole semicircles were analyzed to differentiate and understand the dominant loss mechanisms, including conductive hopping, Debye relaxation or both, using the following equation:

$${\left(\varepsilon \prime \prime \right)}^{2\;}+{\left(\varepsilon^{\prime }-\;\frac{\varepsilon s+\;\varepsilon_{\infty }}{2}\right)}^{2}\;=\;{\left(\;\frac{\varepsilon s-\;\varepsilon_{\infty }}{2}\right)}^{2}$$

**Figure 5:**
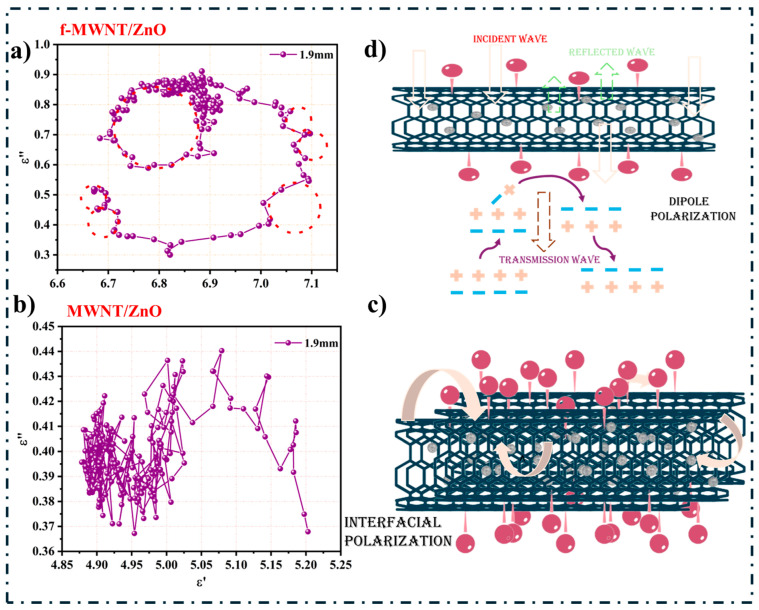
Cole–Cole plots of (**a**) f-MWNT/ZnO and (**b**) MWNT/ZnO vitrimeric composites and a pictorial representation of the polarization mechanisms (**c**,**d**).

Figure 5a demonstrates Cole–Cole plots for the f-MWNT/ZnO composite at 1.9 mm; the plot highlights six distinct semicircular loops representing Debye relaxation processes. These loops indicate that energy dissipates via the relaxation of dipoles and interfacial charges, not solely through electrical conduction [46]. These semicircular arcs indicate the co-existence of various polarization relaxation processes, highlighting the role of dielectric polarization in analyzing absorption behavior. In general, the redistribution of the interfacial charges generates an internal electric field, which aids the separation of charges under an alternating electromagnetic field and originating strong interfacial polarization [47,48]. Alternatively, the plot for MWNT/ZnO in Figure 5b shows a collapsed, disordered distribution of points within a narrow permittivity range. The absence of semicircular arcs indicates that the composites’ dielectric behavior arises from ohmic losses or random electron hopping in the carbon network rather than coordinated polarization relaxation [49].

**Figure 6:**
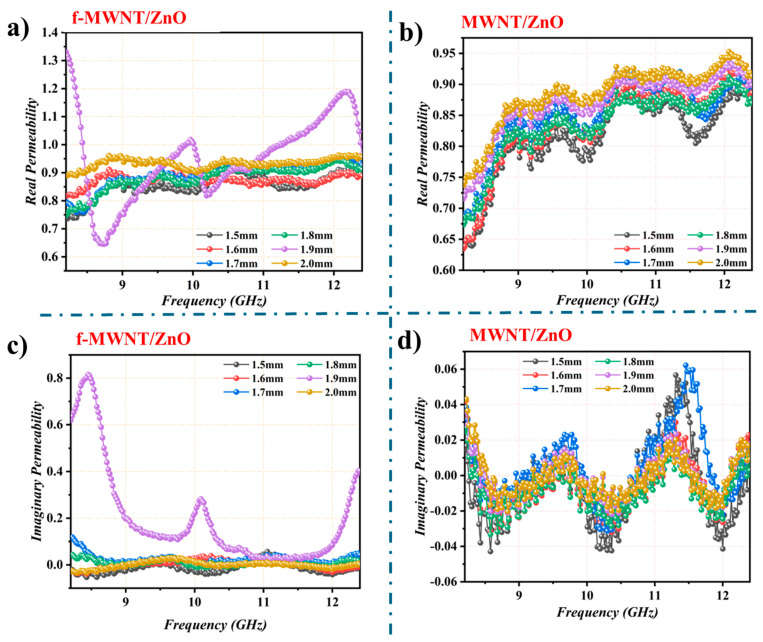
Real permeability curves of (**a**) f-MWNT/ZnO and (**b**) MWNT/ZnO vitrimeric composites. Imaginary permeability curves of (**c**) f-MWNT/ZnO and (**d**) MWNT/ZnO vitrimeric composites.

**Figure 7:**
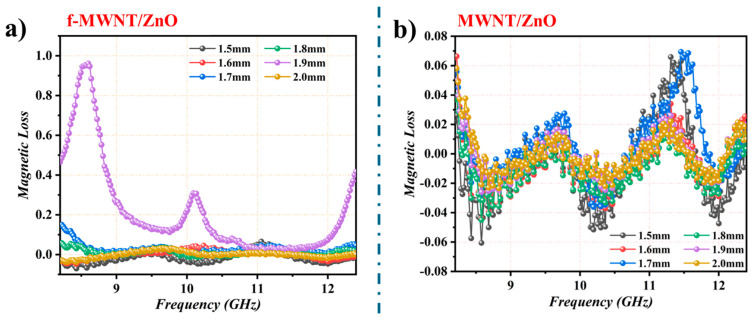
Magnetic loss spectra of (**a**) f-MWNT/ZnO (**b**) MWNT/ZnO vitrimeric composites.

To understand the magnetic response of both composites, the permeability (µ′, µ″) spectra across the X-band was analyzed. Figure 6a,b demonstrate the real permeability (µ′) graphs of f-MWNT/ZnO and MWNT/ZnO composites, where µ′ indicates the material’s ability to store the magnetic energy of electromagnetic waves [24]. The MWNT/ZnO composite shows a steadily increasing trend of permeability; meanwhile, the f-MWNT/ZnO composite at 1.9 mm shows higher fluctuating values in various regions. This indicates that functionalized nanotubes exhibit a better magnetic response than non-functionalized nanotubes. The multiple peaks formed in the entire region correspond to natural resonance at lower frequencies and exchange resonance at higher frequencies [50]. Furthermore, Figure 6c,d show imaginary permeability (µ″) graphs, which show the dissipation of the magnetic response. Prominent resonance peaks are observed for the 1.9 mm f-MWNT/ZnO composite at 8.5 GHz, 10.1 GHz and 12.3 GHz. In contrast, for MWNT/ZnO composites, the peaks hover near zero or negative values, indicating that in an alternating electric field the material generates a magnetic field which radiates the incident EMWs, resulting in negative values [51,52]. Hence, it is the motion of the charges that radiates the magnetic energy out of the composite system, making µ″ negative [53,54]. To further quantify the magnetic response, tan δ_u_ = µ″/µ′ was computed. As shown in Figure 7b, the MWNT/ZnO composites exhibit resonant behavior, characteristic of carbonaceous composites. Conversely, the f-MWNT/ZnO composite in Figure 7a represents a more structured magnetic response, owing to cohesive interfaces formed by the acid functionalization of MWNTs. These interfaces can influence magnetic field distribution, thereby increasing the exchange and natural resonance mechanisms at higher frequencies [55]. Taken together, it can be understood as a synergistic outcome of the dielectric–magnetic response, which is induced by acid functionalization.

The magnetic loss values for f-MWNT/ZnO fluctuate with higher amplitudes, suggesting that the functionalized interfaces act as centers for damping magnetic energy. The eddy current behavior was analyzed using the following equation:

$$C_{o}=\;\frac{\mu \prime \prime }{{\left(\mu \prime \right)}^{2}f}$$

**Figure 8:**
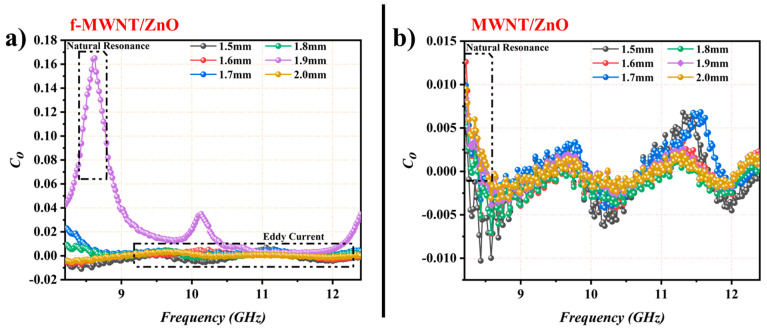
Eddy current curves of (**a**) fMWNT/ZnO and (**b**) MWNT/ZnO vitrimeric composites.

As per electromagnetic theory, if the value remains constant in the frequency range, then the loss contribution is from eddy currents; here, it originates from the interaction of the magnetic field with conductive loops formed by nanotubes [56]. In Figure 8a, eddy current plots are shown for f-MWNT/ZnO composites. The graph shows significant peaks at 8.5 GHz and 10.2 GHz for the 1.9 mm composite, indicating that eddy current loss is not the sole contributor to the dissipation of energy. Probably, the large conductive network formed by the nanotubes is disrupted by numerous interfaces, which reduces eddy current losses and shifts the loss mechanism towards resonance losses [57,58]. On the contrary, MWNT/ZnO composites in Figure 8b show less intense resonance behavior, which might be due to fewer interfaces in the pristine nanotubes.

The absorption efficiency of the composite is quantified by calculating the reflection loss (RL) values.

$$RL=20\log.\left|\frac{Z_{\mathrm{in}}-Z_{0}}{Z_{\mathrm{in}}+Z_{0}}\right|$$

where Z_0_ is the impedance of free air, and Z_in_ can be calculated by using the following equation:

$$Z_{in}=Z_{0}\sqrt{\frac{{µ}_{r}}{\varepsilon_{r}}}\tanh\left(\frac{j2\pi fd\sqrt{{µ}_{r}\varepsilon_{r}}}{c}\right)$$

**Figure 9:**
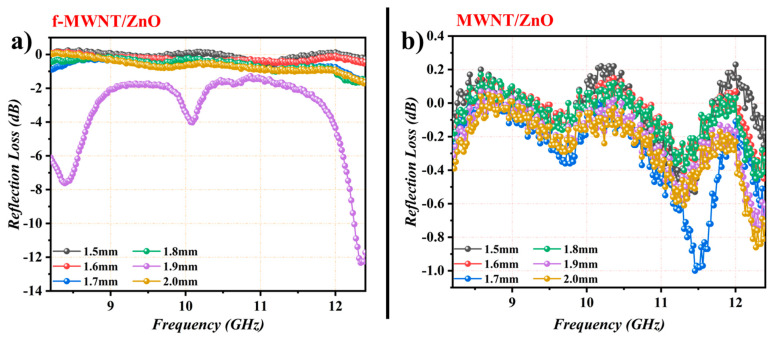
Reflection loss curves of (**a**) f-MWNT/ZnO and (**b**) MWNT/ZnO vitrimeric composites.

Here, µ_r_ represents complex permeability and ε_r_ signifies the complex permittivity of the absorber composite. An RL of −10 dB demonstrates that the material can absorb 90% of the incident electromagnetic wave, 99% if it is −20 dB, and so on [59]. As shown in Figure 9a, acid-functionalized composites show superiority in absorption over pristine MWNT/ZnO composites (Figure 9b). At 9.5 GHz, the 1.9 mm composite f-MWNT/ZnO shows a significant dielectric loss tangent peak, but the µ″ stays low. Similarly, magnetic loss tangent maxima were observed at 8.5, 10 and 12.3 GHz, showing improved dissipation behavior. However, at 8.5 and 10 GHz, the ε″ was low, hindering the effective trade-off between the dielectric and magnetic response, resulting in restricted absorption performance. At 12.3 GHz, a balance between µ″ and ε″ values was observed, generating a powerful synergistic effect. This coordinated interaction facilitates impedance matching and attenuation capabilities, ultimately leading to an RL of −12.7 dB absorption at 12.3 GHz.

To further validate the observed behavior, the matching thickness for the f-MWNT/ZnO composite was evaluated using a quarter-wavelength model via the following equation:

$$t_{m}=\;\frac{nc}{4f\;\sqrt{\left|\varepsilon_{r}\right|\left|\mu_{r}\right|}}\;\left(n=1,3,5,7\ldots \ldots ..\right)$$

**Figure 10:**
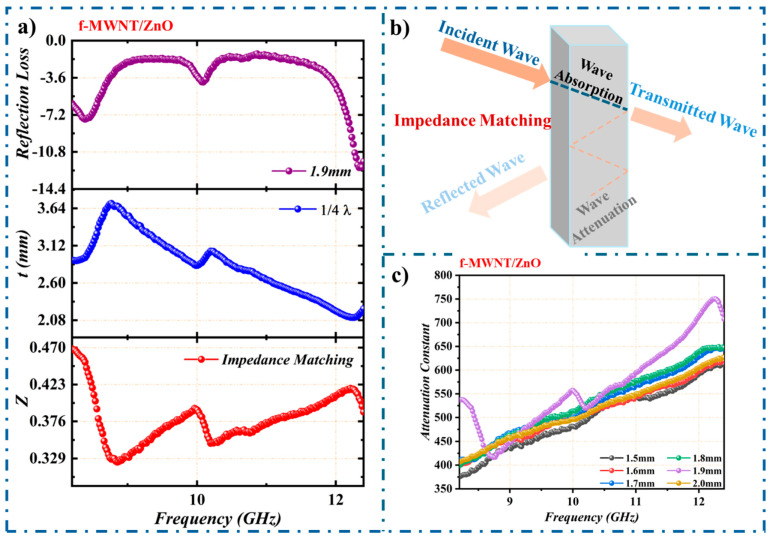
(**a**) Quarter-wavelength, impedance matching and reflection loss relationship; (**b**) impedance matching schematics; (**c**) attenuation constant data of f-MWNT/ZnO vitrimeric composites.

The experimentally obtained RL peak for the 1.9 mm f-MWNT/ZnO composite in Figure 10a aligns well with the theoretical curve, demonstrating the mutual cancellation of the phase between the reflected waves at the air–absorber interface [60]. On the contrary, the MWNT/ZnO composite exhibits significantly weaker RL values and broader, shallower peaks across the X-band. The inferior performance of MWNT/ZnO stems from its lower dielectric loss and poor impedance matching; specifically, the lack of functional groups on the acid-functionalized nanotubes prevents the formation of an effective dissipative network, causing a large portion of the incident waves to be reflected rather than absorbed. Furthermore, the attenuation constant (α) can be computed as follows:

$$\alpha =\sqrt{\left(µ\varepsilon -\varepsilon^{\prime }{µ}^{\prime }\right)+\;\sqrt{{\left({\varepsilon µ}^{\prime }+µ\varepsilon^{\prime }\right)}^{2}+{\left(µ\varepsilon -{µ}^{\prime }\varepsilon^{\prime }\right)}^{2}\;}}\frac{\;\sqrt{2}.\pi .f}{c}$$

α indicates the inherent capability of a composite to disperse EM energy. The stronger the attenuation constant, the larger its ability to attenuate. As shown in Figure 10c, the attenuation constant shows a clear rising trend for all thicknesses; in addition, for the 1.9 mm composite, there are certain peaks as well. These peaks show better attenuation efficacy at such frequency points due to the synergy between carbonaceous and metallic oxide fillers. All of the obtained results systematically explain the obtained behavior of the composites, which includes enhanced polarization losses caused by the acid-functionalization of the nanotubes.

**Figure 11:**
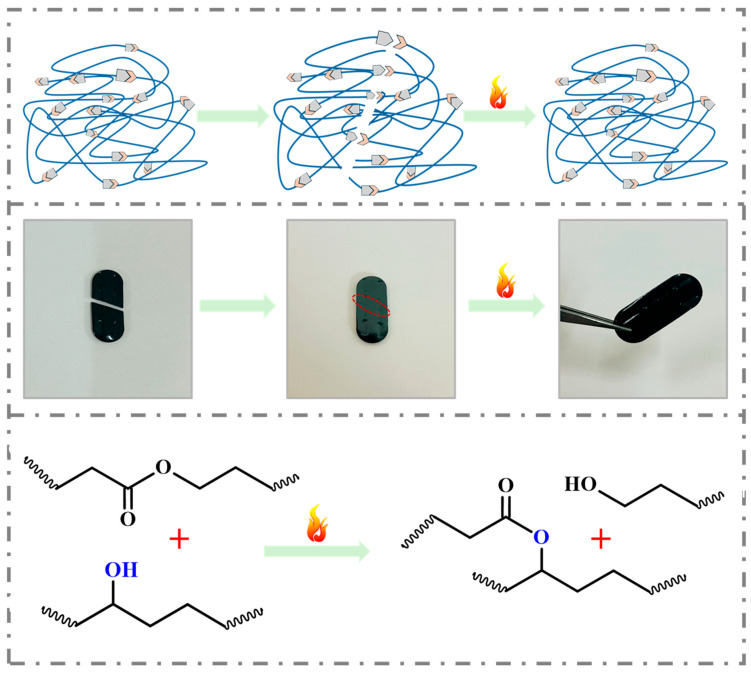
Pictorial and schematic diagram of self-healing mechanism in f-MWNT/ZnO vitrimeric composites.

The self-healing ability of the prepared f-MWNT/ZnO composites is demonstrated in Figure 11 with complete reintegration of the two halves within 40 min at 70 °C. The complete recovery of the segments is due to dynamic transesterification exchange occurring within the matrix, facilitating dissociative bond exchange [61,62,63,64]. At this temperature, the increased chain mobility allows the two distinct segments to rejoin effectively into a single cohesive material. The ability of the composite to achieve complete rejoining in such a short span highlights the importance of these covalent adaptable networks for future engineering applications. Furthermore, thermo-mechanical analysis was carried out for the 1.9 mm f-MWNT/ZnO composite before and after the healing to quantitatively evaluate the restoration of the materials’ properties, as shown in Figure S2. The storage modulus is the elastic stiffness, i.e., the ability of a material to resist permanent deformation and maintain structural integrity under applied load. Therefore, recovery after healing provides insights into the restoration of mechanical performance. The healed f-MWNT/ZnO composite demonstrates ~88% recovery of its properties, validating the dynamic vitrimeric network’s ability to restore the mechanical efficiency.

The comparison Table 1 thoroughly demonstrates the need for a versatile EM wave absorber with low thickness, strong absorption, and reduced filler concentration to make it more suitable for on-ground applications. Additionally, it would be beneficial to have self-healing ability in the coating, which is missing in all the composites listed below, except this work.

## 4. Conclusions and Foresight

To sum, acid-functionalized nanotubes demonstrate better EM absorption performance compared to their non-functionalized counterparts. Preliminary structural analysis confirmed that the intrinsic phases have not been altered, functionalization has significantly improved the interfacial compatibility. The enhanced dielectric response and multiple Debye relaxation processes highlight the critical role of oxygen functional groups in promoting polarization losses. Simultaneously, the improved magnetic response has created dielectric–magnetic synergy. The overall effects led to improved impedance matching and attenuation capability, resulting in an RL of −12.7 dB at 12.3 GHz for the 1.9 mm f-MWNT/ZnO composite. On the contrary, the MWNT/ZnO composites exhibit weaker absorption performance, highlighting the importance of interfacial engineering in such hybrid systems. In addition, the f-MWNT/ZnO composites demonstrate complete reintegration of two halves in 40 min at 70 °C owning to transesterification bond exchange.

For further enhancements, a slight increase in filler concentration while keeping the thickness the same can be carried out to increase the absorption performance. Furthermore, controlling the surface chemistry and defect engineering to create heterogeneous interfaces might give finer polarization effects. Additionally, amalgamating the various fillers with a composite architecture can pave the way for absorption via composites with reduced thickness and filler loadings.

## Figures and Tables

**Table 1:** Comparison of different EM-absorbing composites in X-band.

X-Band Absorber	Reinforcing Components	Thickness (in mm)	Reflection Loss (dB)	References
PA/MWNT	Polyacetylene@MWNT (40 wt% in wax)	2.5	−20.7	[65]
AACNT/BNR	Aligned amorphous carbon nanotube@BaFe_12_O_19_ nanorods (70 wt%)	2.0	−21.5	[66]
BaCo_x_Mn_x_Ti_x_Fe_12−4x_O_4_	Modified Barrium ferrite (90%)	2.0	−14.7	[67]
BaFe_12_O_19_ + BaTiO_3_	(BaFe_12_O_19_ + BaTiO_3_)/Polyaniline	2.0	−10.4	[68]
BaTiO_3_/PA	BaTiO_3_-Polyaniline (25:75)	2.98	−15	[69]
G/Fe_3_O_4_@Fe/ZnO	Graphene/Fe_3_O_4_@Fe-coreshell nanoparticles/ZnO	5.0	−38.4	[70]
Fe/Al_2_O_3_	Fe (5 vol%)/Al_2_O_3_ (94 vol%)	1.7	−15	[71]
Fe_3_O_4_/PPy/CNT	Fe3O4/Polypyrrole/carbon nanotubes	3.0	−25.9	[72]
f-MWNT/ZnO	Acid-functionalized carbon nanotubes (4 wt%)/ZnO (20 wt%)	1.9	−12.7	This work

## Data Availability

The original contributions presented in this study are included in the article. Further inquiries can be directed to the corresponding author.

## Supplementary Materials

The following supporting information can be downloaded at: https://www.mdpi.com/article/10.3390/polym18111374/s1, Figure S1: (a) SEM image and (a′) EDX mapping of MWNT/ZnO composite; Figure S2: (a) Storage modulus and (b) Loss modulus graphs of 1.9mm f-MWNT/ZnO composite before and after healing.
