# Supplementary material for: Interfacial Polarization-Driven Dielectric–Magnetic Synergy in Vitrimeric f-MWNT/ZnO Composites: Effect of MWNT Functionalization
Source: Polymers (Basel). 2026 Jun 1;18(11):1374. doi: 10.3390/polym18111374 (PMC13258962; doi:10.3390/polym18111374)
Supplement: Supplementary file 1 [file polymers-18-01374-s001.zip › polymers-4321211-supplementary.pdf]

# Interfacial Polarization-Driven Dielectric–Magnetic Synergy in Vitrimeric f-MWNT/ZnO Composites: Effect of MWNT Functionalization

**Nehal Kaushik [1], Madhuri Surya [1], Divyanshi Nautiyal [1], Rajkumar Patel [2,*] and Sravendra Rana [1,*]**

[1] School of Engineering, University of Petroleum & Energy Studies (UPES), Dehradun-248007, Uttarakhand, India; nehal.110757@stu.upes.ac.in (N.K.); madhuri.130296@stu.upes.ac.in (M.S.); divyanshi.18909@stu.upes.ac.in (D.N.)

[2] Integrated Science and Engineering Division, Underwood International College, Yonsei University,
Incheon 21983, Republic of Korea

* Correspondence: rajkumar@yonsei.ac.kr (R.P.); srana@ddn.upes.ac.in (S.R.)

SEM and EDX of MWNT/ZnO vitrimeric composite

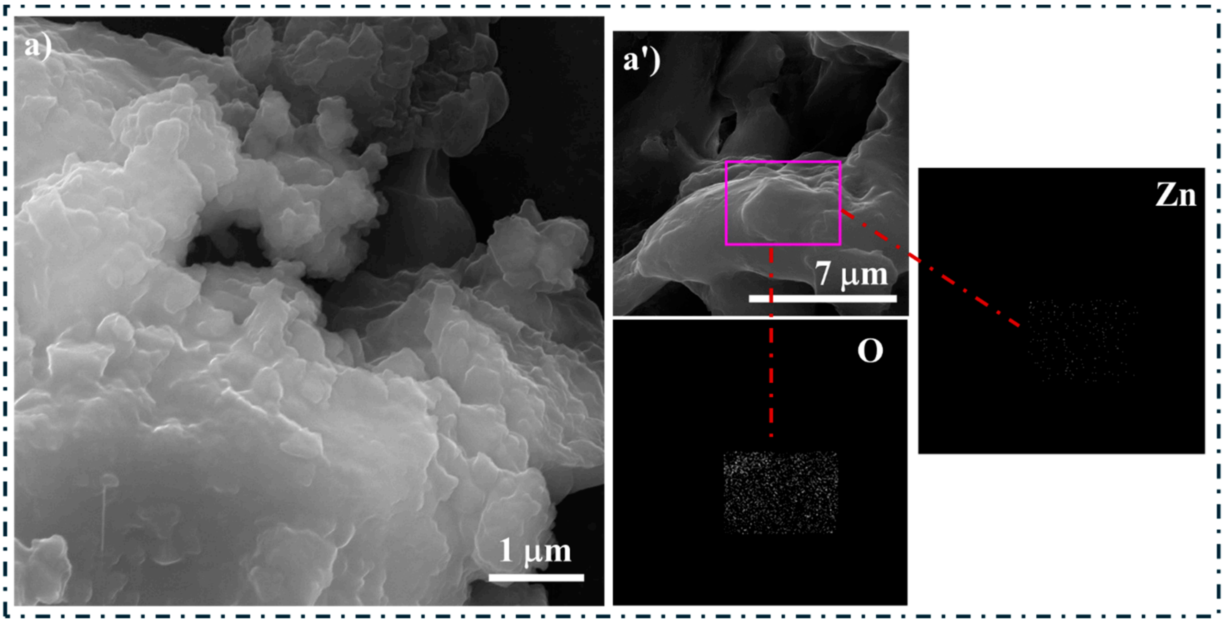


**Figure S1.** (**a**) SEM image and (**a'**) EDX mapping of MWNT/ZnO composite.

Thermo-mechanical analysis of f-MWNT/ZnO vitrimeric composite

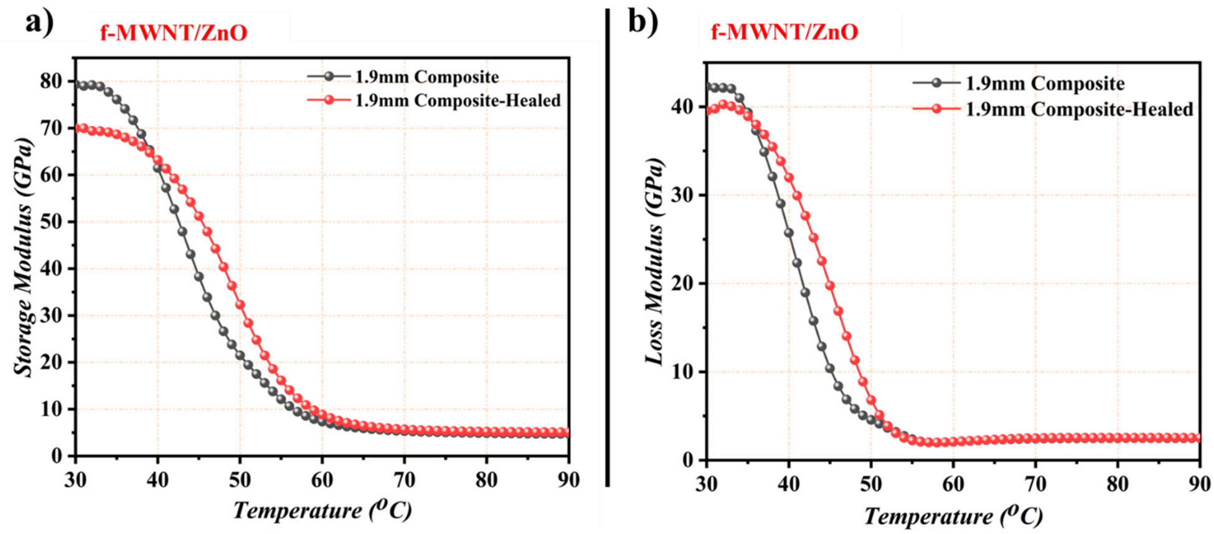


**Figure S2.** (**a**) Storage modulus and (**b**) Loss modulus graphs of 1.9mm f-MWNT/ZnO composite before and after healing.
